# LSTR Technique With CTZ Antibiotic Paste in Primary Molar: 5-Year Follow-Up Case Report

**DOI:** 10.1155/crid/8629672

**Published:** 2025-11-09

**Authors:** María del Carmen Sánchez Pumeda, Olga Henríquez

**Affiliations:** Department of Pediatric Dentistry, Universidad Nacional Pedro Henríquez Ureña, Santo Domingo, Dominican Republic

**Keywords:** case report, CTZ antibiotic paste, LSTR, primary molar, pulp therapy

## Abstract

Reports describing long-term outcomes of the lesion sterilization and tissue repair (LSTR) technique in primary molars are limited. This paper presents a distinctive case in which a primary molar with complete root development and furcation involvement—usually an indication for conventional pulpectomy—was instead treated with LSTR, a technique generally recommended in advanced root resorption. The case adds evidence on the potential indications and clinical versatility of this approach in pediatric dentistry. A pediatric patient with uncooperative behavior presented with discomfort in a primary molar. Clinical and radiographic examination revealed pulp necrosis, complete root formation, and a furcation lesion. Although pulpectomy was the conventional indication for such findings, LSTR was chosen as a conservative alternative. Treatment consisted of the placement of an antibiotic mixture in the pulp chamber to disinfect and sterilize the root canal system without mechanical instrumentation, with the objective of controlling infection and promoting tissue repair. At the 5-year follow-up, the tooth remained functional and symptom-free, with radiographic evidence of healing and tissue regeneration, demonstrating favorable long-term outcomes. This case shows that LSTR can provide favorable long-term outcomes even in situations where pulpectomy would traditionally be indicated. It supports LSTR as a less invasive and effective option for preserving primary molars until their natural exfoliation.

## 1. Introduction

Despite advances in preventive strategies, dental caries remains a major public health problem in developing countries, significantly affecting the quality of life of pediatric patients [1]. Once the disease progresses to the pulp tissue in primary teeth, treatment becomes complex. Pulpectomy, although recommended by the American Academy of Pediatric Dentistry (AAPD), is often challenging due to the peculiar morphology of primary roots—tortuous canals, multiple accessory canals, atypical resorption—and the frequent difficulties in behavior management during treatment [2, 3]. In 2020, the AAPD incorporated for the first time the lesion sterilization and tissue repair (LSTR) technique as an alternative for cases where pulpectomy is not feasible due to advanced root resorption [4]. Unlike pulpectomy, LSTR does not require canal instrumentation; only the coronal pulp tissue is removed, and an antibiotic mixture is placed to disinfect and sterilize the root canal system [4]. One of the most widely studied combinations is the CTZ paste, composed of chloramphenicol, tetracycline, and zinc oxide–eugenol, which has demonstrated antimicrobial activity against *Streptococcus aureus*, *Enterococcus faecalis*, *Pseudomonas aeruginosa*, *Bacillus subtilis*, and *Candida albicans* [5]. Studies have shown favorable clinical and radiographic outcomes [6, 7], biocompatibility comparable to calcium hydroxide [8], absence of alterations in alveolar blood cells [9], and no evidence of damage to the permanent successor tooth [10].

What makes this case unique is that a primary molar with complete root formation and furcation involvement—classically indicated for conventional pulpectomy—was treated with the LSTR technique using CTZ paste. In addition, the case was followed longitudinally for 5 years, providing rare evidence on the long-term effectiveness of this conservative treatment, as well as the absence of alterations in the enamel of the permanent successor.

The aim of this article is to report the clinical and radiographic outcomes of this 5-year follow-up, highlighting the potential of LSTR with CTZ paste as a less invasive and effective alternative for pulp therapy in pediatric dentistry.

## 2. Case Presentation

**Figure 1:**
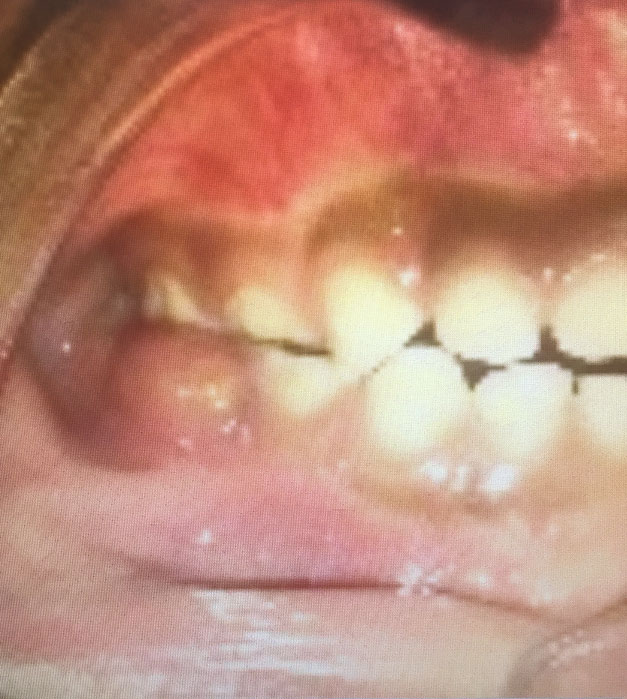
Localized edema in the buccal area of Tooth 85, indicative of an abscess.

**Figure 2:**
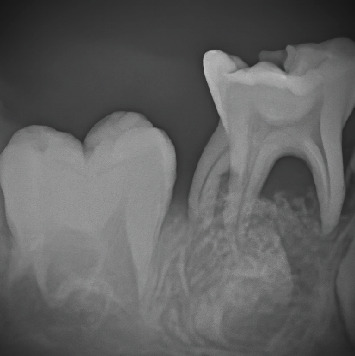
Periapical radiograph of Molar #85, where radiolucency in the furcation and an extremely deep carious lesion is observed.

**Figure 3:**
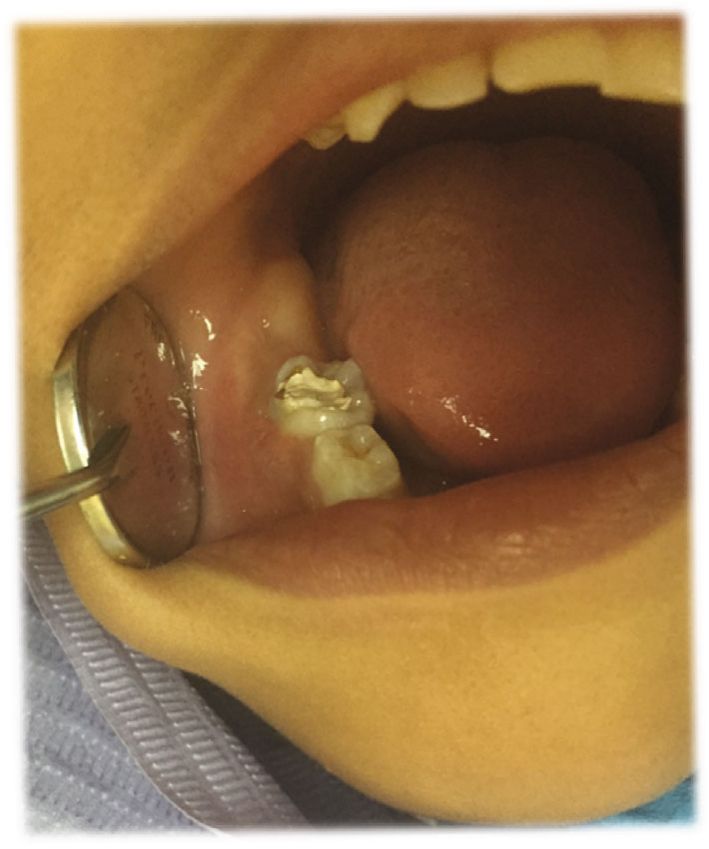
Two weeks after treatment, where the absence of the abscess is observed.

**Figure 4:**
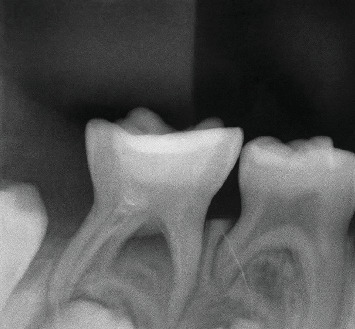
Two weeks after treatment, the furcation radiolucency has decreased and increased radiopacity is observed.

**Figure 5:**
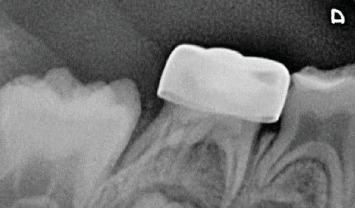
After 2 months, a significant reduction in furcation radiolucency is observed. The apparent crown misfit is an effect of the image perspective and does not reflect the true clinical result.

**Figure 6:**
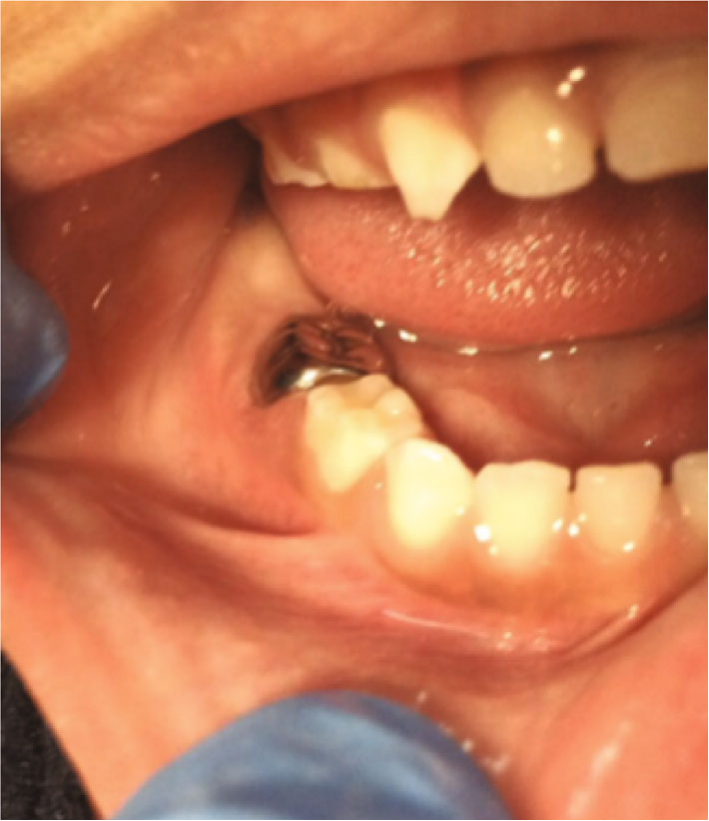
After 2 months of follow-up, where the absence of lesion is observed.

**Figure 7:**
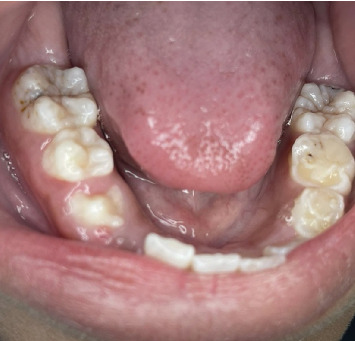
Lower occlusal image showing the presence of the clinical crown of the lower second Premolar #45 without clinical alteration of the enamel.

A 5-year-old female patient presented in March 2017 to the pediatric dentistry department with discomfort in the lower mandibular region and localized edema in the buccal area of Tooth 85, indicative of an intraoral abscess (Figure 1). The patient exhibited uncooperative behavior according to the Frankl Behavior Scale (–), characterized by fear and lack of cooperation during the dental visit. The patient had no relevant medical history. No significant family history related to oral or systemic diseases was reported. No relevant psychosocial factors were identified, and no genetic conditions associated with dental anomalies were noted. The patient had no history of previous dental or medical interventions relevant to the present condition. Intraoral examination revealed an active and deep carious lesion on the occlusal surface of Tooth 85, with localized swelling in the buccal region. Radiographic evaluation demonstrated an extremely deep carious lesion in direct contact with the pulp tissue, roots without resorption, and a large radiolucency in the furcation region (Figure 2). No laboratory tests were required for this case. The main diagnostic challenge was related to the patient's negative behavior, which complicated the clinical examination and radiographic procedures. Despite these difficulties, sufficient diagnostic information was obtained to establish an accurate diagnosis. Based on the clinical and radiographic findings, a diagnosis of pulp necrosis in Tooth 85 was established. Differential diagnoses considered included irreversible pulpitis and acute dentoalveolar abscess, but these were excluded due to the extent of the necrotic tissue and radiographic presentation. The prognosis was considered favorable for maintaining the tooth in function until its natural exfoliation, provided that infection control was achieved and regular follow-up was maintained. Treatment consisted of the LSTR technique using CTZ paste. The procedure included a pulpotomy without removal of the radicular pulp. Topical anesthesia (benzocaine 20%) was applied for 2 min, followed by the truncal technique with 2% lidocaine, also left to act for 2 min. Absolute isolation was achieved, and the access opening was performed. A 2% sodium hypochlorite solution was applied for 1 min. On a sterile tile, a mixture of tetracycline 500 mg, chloramphenicol 500 mg, and zinc oxide–eugenol cement was prepared and placed in the pulp chamber, followed by provisional restoration with IRM cement. No modifications to the planned therapeutic intervention were required during treatment or follow-up. At the 2-week recall, the abscess had disappeared (Figure 3), and radiographs showed a reduction in furcation radiolucency (Figure 4). A stainless steel crown was placed. Clinical and radiographic follow-ups at 2 months (Figures 5 and 6), 6 months, and 1 year confirmed continued healing. At the 5-year evaluation, the tooth exfoliated naturally without complications, and no alterations were observed in the permanent successor (Figure 7). The patient attended all scheduled follow-up visits, and the intervention was well tolerated without any signs of discomfort or rejection. No adverse or unanticipated events occurred during or after the intervention.

## 3. Discussion

This case report documents the successful use of the LSTR technique with CTZ antibiotic paste in a primary molar, with a 5-year follow-up. The intervention proved to be effective for the resolution of the abscess, with preservation of the affected tooth and absence of clinical signs and symptoms until exfoliation. This case also reflects the clinical applicability of the LSTR technique in resource-limited settings and highlights the importance of integrating conservative alternatives such as LSTR into pediatric dentistry training and practice in developing countries.

The LSTR technique, based on disinfection of the lesion and tissue repair, has been proposed as an alternative in cases where the primary molar cannot undergo a conventional pulpectomy due to advanced root resorption [4]. In this case, the primary molar exhibited clear indications for a conventional pulpectomy, as the roots were fully developed and a furcation lesion was present. Under standard guidelines, these findings would justify pulpectomy as the treatment of choice. Nevertheless, an alternative approach was taken with the use of LSTR. According to the AAPD, LSTR is generally reserved for advanced cases of root resorption, where pulpectomy may no longer be feasible due to insufficient remaining root structure [4]. Interestingly, despite the fact that this tooth did not meet the typical criteria for LSTR, the treatment achieved satisfactory clinical and radiographic outcomes. This contrast between the expected indications outlined by the AAPD and the successful results obtained underscores the potential flexibility of LSTR as a therapeutic option.

A randomized clinical study with a 36-month follow-up demonstrated no statistically significant differences in clinical and radiographic outcomes between pulpectomy and LSTR, recommending that the Academy reviews its guidelines in order to provide greater comfort and reduce treatment time for pediatric patients [11].

With respect to the components of antibiotic pastes, the AAPD recommends avoiding tetracycline derivatives [4], due to possible adverse effects in children. Traditionally, tetracyclines have been associated with tooth discoloration and defects in enamel formation when administered systemically during the period of odontogenesis [12, 13]. However, within the framework of the LSTR technique, tetracycline is applied topically and locally, in very small amounts and strictly confined to the pulp chamber, which markedly reduces the likelihood of systemic absorption. Available clinical evidence and several reviews have not documented any changes in the pigmentation or structure of the permanent successor tooth following the use of CTZ paste in primary molars [7, 9, 14]. According to Moura et al., current warnings regarding the use of tetracyclines in this context are mainly extrapolated from the risks associated with systemic administration, rather than derived from specific clinical data indicating actual adverse effects [14].

From a risk–benefit perspective, it is essential to consider that the conventional alternative, pulpectomy, typically involves longer procedures and greater patient cooperation. These factors pose particular challenges in young or uncooperative pediatric patients, in whom the likelihood of clinical failure and the need for general anesthesia increase. In contrast, the controlled and localized use of tetracycline in the LSTR technique offers relevant clinical advantages: effective infection control, preservation of the tooth in the arch, and reduced procedural complexity, with no reports to date of adverse effects on permanent teeth or systemic health [7, 9, 11–14]. Nevertheless, it is emphasized that this technique should be indicated with sound clinical judgment, adhering to appropriate proportions and avoiding indiscriminate use.

Another factor contributing to the success of the technique is that antibiotic mixtures should be prepared on the same day of the procedure and in the correct proportions to ensure fresh antimicrobial potential [15]. In this case, the antibiotics were crushed and prepared at the time of the appointment. In addition, the hermetic sealing of the molar with a stainless steel crown may have enhanced the success of treatment, since this type of restoration has proven superior to conventional restorations by offering better marginal adaptation and preventing leakage [14].

The main strength of this case is the 5-year longitudinal follow-up, which provides rare evidence of the long-term effectiveness of LSTR with CTZ paste in a primary molar. Another strength is that the patient exhibited uncooperative behavior, highlighting the value of LSTR in clinical scenarios where pulpectomy may be challenging. The use of a stainless steel crown and the fresh preparation of the paste further contributed to the favorable outcome. However, this case report also has limitations. Being a single case, the results cannot be generalized to all pediatric patients. The absence of a direct comparison with pulpectomy in the same clinical context limits the ability to establish equivalence between both treatments. Finally, the use of tetracycline remains controversial, as international guidelines express caution regarding its use, despite the absence of strong evidence of adverse effects in pediatric patients.

## 4. Conclusion

The LSTR technique with CTZ paste proved to be a conservative and effective treatment option for a primary molar with pulp necrosis, achieving 5 years of asymptomatic function, radiographic healing, and natural exfoliation without affecting the permanent successor. This case highlights that LSTR can be considered a practical alternative to conventional pulpectomy, particularly in children with difficult behavior or in settings with limited resources, and underscores the need to re-examine current guidelines on its indications.

## Patient Perspective

The child's mother reported that the treatment was well accepted despite the initial fear and lack of cooperation during the dental visit. She highlighted that the procedure was shorter and less invasive than she had anticipated. During the follow-up appointments, she expressed satisfaction with the absence of pain, swelling, or additional dental problems. At the end of the 5-year follow-up, she considered the treatment successful, as the primary tooth exfoliated naturally and the permanent successor erupted without complications.
